# Genotypes and haplotypes of the methyl-CpG-binding domain 2 modify breast cancer risk dependent upon menopausal status

**DOI:** 10.1186/bcr1283

**Published:** 2005-07-19

**Authors:** Yong Zhu, Heather N Brown, Yawei Zhang, Theodore R Holford, Tongzhang Zheng

**Affiliations:** 1 Department of Epidemiology and Public Health, Yale University School of Medicine, New Haven, Connecticut, USA

## Abstract

**Introduction:**

*MBD2*, the gene encoding methyl-CpG-binding domain (MBD)2, is a major methylation related gene and functions as a transcriptional repressor that can specifically bind to the methylated regions of other genes. MBD2 may also mediate gene activation because of its potential DNA demethylase activity. The present case-control study investigated associations between two single nucleotide polymorphisms (SNPs) in the *MBD2 *gene and breast cancer risk.

**Methods:**

DNA samples from 393 Caucasian patients with breast cancer (cases) and 436 matched control individuals, collected in a recently completed breast cancer case–control study conducted in Connecticut, were included in the study. Because no coding SNPs were found in the *MBD2 *gene, one SNP in the noncoding exon (rs1259938) and another in the intron 3 (rs609791) were genotyped. Odds ratios (ORs) with 95% confidence intervals (CIs) were calculated to estimate cancer risk associated with the variant genotypes and the reconstructed haplotypes.

**Results:**

The variant genotypes at both SNP loci were significantly associated with reduced risk among premenopausal women (OR = 0.41 for rs1259938; OR = 0.54 for rs609791). Further haplotype analyses showed that the two rare haplotypes (A-C and A-G) were significantly associated with reduced breast cancer risk (OR = 0.40, 95% CI = 0.20–0.83 for A-C; OR = 0.47, 95% CI = 0.26–0.84 for A-G) in premenopausal women. No significant associations were detected in the postmenopausal women and the whole population.

**Conclusion:**

Our results demonstrate a role for the *MBD2 *gene in breast carcinogenesis in premenopausal women. These findings suggest that genetic variations in methylation related genes may potentially serve as a biomarker in risk estimates for breast cancer.

## Introduction

It has recently been recognized that cancer is a manifestation of both abnormal genetic and epigenetic events [1]. Dysregulated epigenetic controls, which usually are represented by abnormal DNA methylation patterns such as global hypomethylation and region specific hypermethylation, are a hallmark of most cancers. Although the precise mechanisms underlying methylation alterations are far from being fully understood, the overall methylation process is mainly regulated by several groups of regulatory proteins [2-4].

The methyl-CpG-binding domain (MBD) proteins are among these protein families that bind specifically to a methylated gene and mediate transcriptional repression via effects on chromatin structure. Thus far, five MBD genes have been identified in mammalian cells that encode putative MBDs, namely *MeCP2*, *MBD1*, *MBD2*, *MBD3*, and *MBD4 *[5-7]. Human MBD genes are considered housekeeping genes because they are widely expressed in somatic tissues [6]. Given the epigenetic role of MBD proteins in regulating gene expression, MBDs may be involved in cancer development by affecting the expression of cancer related genes. In fact, there is growing evidence that aberrant expression of MBD proteins is associated with human cancers [8,9].

The *MBD2 *gene is mapped to the conserved region within human chromosome 18q21 [10]. Genomic sequence analysis determined that the *MBD2 *gene contains six exons and one noncoding exon spanning more than 50 kb in the genome. The *MBD2 *gene encodes two potential forms of protein MBD2 that correspond to the initiation of translation starting at either the first (MBD2a; 43.5 kDa) or second (MBD2b; 29.1 kDa) methionine codons. The signal functions of MBD2 are to bind specifically to methylated gene promoters and recruit histone deacetylases and chromatin remodeling proteins. The altered chromatin structure resulting from the binding of these factors may be resistant to the transcriptional machinery and, as a result, repress gene expression [11,12]. A recent finding also suggests that MBD2 has potential DNA demethylase activity [13], implying that it might mediate gene activation in addition to transcriptional repression. However, two subsequent studies could not demonstrate any demethylase activity of MBD2 [14,15], and this inconsistency in the functions of MBD2 remains to be resolved.

Although our understanding of the exact function of MBD2 in epigenetics is still in its early stages, several studies in human cancer research have demonstrated that the MBD2 protein plays a role in tumorigenesis. For example, a recent study [16] showed that breast carcinomas exhibit alterations in MBD2 expression. One interesting finding from that study was that breast carcinomas can be divided into two groups, with one expressing very high levels of MBD2 and the other expressing a much lower level. MBD2 has also been reported to be involved in the repression of GSTP1 transcription in breast cancer cells [17]. Moreover, a significant reduction in MBD2 mRNA expression was found in human colorectal and gastric cancerous tissues [18] and peripheral blood lymphocytes [19] in bladder cancer patients, implying a protective role for MBD2 in tumorigenesis. MBD2 protein expression and its demethylase activity were detected in normal human prostate tissue but not in cancerous tissue [20]. These differences between types of cancers in the abundance of MBD2 levels may reflect different roles for MBD2 either in transcriptional repression or in the demethylation process.

Given that there is a potential role for MBD2 in tumorigenesis, we hypothesized that genetic polymorphisms in the *MBD2 *gene may modify an individual's susceptibility to human cancers. In this molecular epidemiologic study, we genotyped two single nucleotide polymorphisms (SNPs; rs1259938 and rs609791) in the *MBD2 *gene to investigate whether genetic variations in the *MBD2 *gene are associated with breast cancer risk and whether the potential associations are modified by menopausal status.

## Materials and methods

### Study population

This study was built upon a recently completed breast cancer case–control study that was undertaken in Connecticut, USA. Detailed information regarding the study population is provided elsewhere [21]. Briefly, a total of 475 histologically confirmed incident breast cancer cases (ICD-O, 174.0–174.9) and 502 randomly selected control individuals were identified from the Tolland and New Haven County area of Connecticut between 1 January 1994 and 31 December 1997. All of the cases and controls were in the age range 30–80 years and had no previous diagnosis of cancer with the exception of nonmelanoma skin cancer.

For New Haven County, eligible cases were identified from the major hospital of the county (Yale–New Haven Hospital) through the computer database system at the Department of Surgical Pathology. Controls were also randomly selected from the computer database system from among women who were histologically confirmed to be without breast cancer. The participation rates were 77% for cases and 71% for controls in New Haven County. For Tolland County, because there was no major county hospital in this county, newly diagnosed breast cancer cases were identified from area hospital records by the Rapid Case Ascertainment system at the Yale Comprehensive Cancer Center. Controls from Tolland Country were recruited through random digit dialing methods for those under age 65 years and randomly selected from Health Care Financing Administration files for those aged 65 years and over. The participation rates were 74% for cases and 64% for controls in Tolland County.

The study pathologist reviewed all of the pathologic diagnoses for breast cancer patients and benign breast disease controls. Breast carcinoma were classified as carcinoma *in situ*, invasive ductal, or lobular carcinoma, and were staged according to the American Joint Committee on Cancer staging system [22].

### Data collection

Informed consent was obtained from all study participants before collection of epidemiologic data through personal interview. The 45-min in-person interview, completed by all study participants, was administered by trained interviewers following institutional guidelines for human subjects. Data on smoking habits, alcohol consumption, and hormone replacement therapy of case and control individuals was obtained. Other information, including menstrual and reproductive factors (age at menarche, age at first pregnancy, age at menopause, parity, lifetime lactation history), family breast cancer history, lifetime occupational history, body mass index, hair dye use, and residence history, was also collected. Dietary information was obtained using a scannable semiquantitative food frequency questionnaire developed by the Fred Hutchinson Cancer Research Center, designed to optimize estimation of fat intake. Menopausal status was assessed at the time of diagnosis. Women with hysterectomy or bilateral oophorectomy were considered to be postmenopausal women, whereas very few women with dubious menopausal status were considered to represent missing data. At the completion of the interview, blood was drawn for DNA isolation and subsequent molecular analysis. The status of all samples – case or control – was concealed before they were handed to laboratory personnel.

### Single nucleotide polymorphism selection

A SNP search using the National Center for Biotechnology Information SNP database [23] showed no non-synonymous SNPs in the coding region of the *MBD2 *gene. Therefore, two noncoding SNPs were chosen for genotyping. One (rs1259938) is located in the noncoding exon and another (rs609791) is located in intron 3 of the *MBD2 *gene. The noncoding exon is generally found at the 3'-untranslated region of a gene and this is now widely acknowledged. There is increasing evidence indicating that the 3'-untranslated region of a gene plays a vital biologic role in many post-transcriptional regulatory pathways that control mRNA localization, stability, and translation efficiency [24].

### Genotyping methods

The restrictional fragment length polymorphism PCR assay was used to determine the genotypes of SNP rs1259938. The genomic DNA used for the assay was extracted from peripheral blood lymphocytes. The PCR primers used for amplifying this polymorphism were as follows: forward 5'-CCTTGCCTGTGACTTGGACT-3' and reverse 5'-TCGCGAGTTTCAACAGAAAA-3'. Standard PCR was performed in a 25 μl volume with annealing temperature at 58°C and followed by an overnight digestion with *Xba*I (New England BioLabs, Beverly, MA, USA) at 37°C. The products were separated for 45 min at 220 V on a 4% agarose gel stained with ethidium bromide. Following electrophoresis, the homozygous G/G alleles were represented by a DNA band with size at 319 bp, whereas the homozygous A/A alleles were represented by DNA bands with sizes at 103 bp and 216 bp, and the heterozygotes displayed a combination of both alleles (103 bp, 216 bp and 319 bp).

The TaqMan Assay was used to determine the genotypes of SNP rs609791. Assays-on-Demand primers and probes (C_3079439_10; Applied Biosystems, Inc., Foster City, CA, USA) were mixed with PCR reagents following the manufacturer's instructions in the TaqMan assay. Plates were sealed and cycled at 95°C for 5 min, followed by 45 cycles at 92°C for 15 seconds, and 60°C for 1 min in a Stratagene Real-Time Mx3000 thermocycler (Stratagene Corp., La Jolla, CA, USA).

Each genotyping plate contained positive and negative controls. Approximately 5% of the samples were duplicated to ensure quality control in genotyping and two reviewers separately performed genotype scoring to confirm results.

### Statistical analysis

Because more than 90% of the study participants were Caucasians, with about 6% being black, 1% Asian, and 2% other races, we restricted our analysis to Caucasians only (393 cases and 436 controls). Pearson's χ^2 ^test was used to evaluate differences in the distribution of selected characteristics between cases and controls. Genotype frequencies at both SNP loci in the control population were first checked for compliance with Hardy–Weinberg equilibrium using STATA statistical software (StataCorp, LP, College Station, TX, USA). Haplotype estimation was calculated using the PHASE program, which reconstructs haplotypes from population genotyping data [25]. The best haplotypes estimated by the PHASE were assigned to each study participant. STATA was also used to calculate both crude and adjusted odds ratios (ORs). ORs with 95% confidence intervals (CIs) were reported to illustrate relative cancer risk associated with genotypes and haplotypes.

For a SNP genotype, study participants with homozygous common allele were used as the reference group in OR calculation. For haplotype analysis, the most common haplotype (G-C) was used as the reference group in risk estimation. Logistic regression was used to control for confounding by age (as a continuous variable), body mass index (<25 kg/m^2^, 25–29.99 kg/m^2^, >29.99 kg/m^2^), family history of breast cancer in first-degree relatives, family income (tertiles based on distribution of controls), lifetime months of breastfeeding (never, 1–5, 6–15, >15 months) and study site (New Haven County, Tolland County). Control of other variables (such as age at menarche, age at menopause, number of live births) did not change the ORs significantly, and these variables were not included in the final model.

## Results

This study, which included 393 breast cancer cases and 436 controls, was composed entirely of Caucasians. Table 1 presents the distribution of selected baseline characteristics for cases and controls. There were significantly more postmenopausal women in the case population (77.6%) than among controls (66.5%), indicating an increased risk for breast cancer associated with menopausal status. In addition, data showed that more controls had higher family income (31%) compared to the incidence of high family income in breast cancer cases (23.5%). No other baseline factors exhibited a material difference between cases and controls.

The genotype distributions of both SNP loci for cases and controls (Table 2) were in Hardy–Weinberg equilibrium (χ^2 ^= 0.35, *P *= 0.56 for rs1259938; χ^2 ^= 0.31, *P *= 0.57 for rs609791).

Among all women, we found no overall associations between genotypes at these two loci and breast cancer risk after adjustment for age, menopausal status, family history of breast cancer in first-degree relatives, family income, body mass index, lifetime months of breastfeeding, and study site. Among premenopausal women, a reduced breast cancer risk was significantly associated with variant genotypes (homozygous minor allele + heterozygote) at both SNP loci. Specifically, women with G/A and A/A at rs1259938 had 59% reduced breast cancer risk (OR = 0.41, 95% CI = 0.23–0.72) and women with C/G and G/G at rs609791 had 46% reduced breast cancer risk (OR = 0.54, 95% CI = 0.30–0.96). However, no significant associations were detected among postmenopausal women.

These two SNP loci in the *MBD2 *gene may generate four possible haplotypes, and their frequency distributions among cases and controls are shown in Table 3. G-C was the most common haplotype, with a frequency of 66.90% in our control group. The frequencies of the other three haplotypes were 5.56% (G-G), 11.70% (A-C), and 15.84% (A-G) in controls. Among all female participants, none of these haplotypes was associated with breast cancer risk. However, in premenopausal women the two rare haplotypes halved breast cancer risk, with ORs of 0.40 (95% CI = 0.20–0.83, *P *= 0.013) for A-C and 0.47 (95% CI = 0.26–0.84, *P *= 0.011) for A-G. Similar associations were not observed in postmenopausal women.

## Discussion

It is becoming clear that carcinogenesis is a stepwise process of accumulation of both genetic and epigenetic abnormalities that can lead to cellular dysfunction. A large body of evidence has demonstrated that the epigenetic process is involved in breast carcinogenesis by influencing several broad gene categories, including cell cycle regulation, cell growth, steroid receptors, tumor susceptibility, carcinogen detoxification, cell adhesion, and inhibitors of matrix metalloproteinase genes. For example, methylation of p16 promoter and exon 1 regions are observed in both human breast cancer cell lines and 20–30% of primary breast cancers [26,27]. Methylation of the promoter region of *GSTP1*, a member of the glutathione S-transferases, which are a supergene family involved in the detoxification of carcinogens, is associated with gene inactivation in about 30% of primary breast carcinomas [28]. DNA methylation has also been found to be an alternative mechanism of inactivation of *BRCA1 *[29,30], a gene that accounts for one half of inherited breast carcinomas [31]. Moreover, three members of the steroid hormone superfamily, including estrogen receptor, progesterone receptor and retinoic acid receptor, have long been linked to mammary carcinogenesis [32] and recent studies have shown that epigenetic alterations appear to play a role in silencing estrogen receptors and retinoic acid receptors in breast malignancy [33-35]. Given the increasing evidence for a role of the epigenetic process, especially DNA methylation, in breast carcinogenesis, it is speculated that some genetic variations in methylation related genes may affect the expression and function of these genes and consequently contribute to breast cancer development.

Findings from the present study show associations between genotypes and haplotypes of the *MBD2 *gene and breast cancer, which have not previously been examined. These results support a potential role for methylation related genes in breast tumorigenesis. Interestingly, MBD2 polymorphisms have different effects in women depending on menopausal status. Our results demonstrate significant associations between MBD2 genotypes and haplotypes and breast cancer risk in premenopausal women but not in postmenopausal women. In fact, menopausal effects on breast cancer risk have also been observed in a previous study investigating genetic polymorphisms in catechol-*O*-methyltransferase [36]. That study found that the low-activity allele of catechol-*O*-methyltransferase was associated with increased risk among premenopausal women (OR = 2.1, 95% CI = 1.4–4.3) but was inversely associated with postmenopausal risk (OR = 0.4, 95% CI = 0.2–0.7). Our findings support arguments from previous studies suggesting that different etiologies may be involved in breast carcinogenesis between premenopausal and postmenopausal women [36,37].

Although the mechanisms are not elucidated, menopausal effects on the role of MBD2 in breast cancer development may be related to changes in sex hormone levels. One of the phases of breast cancer pathogenesis is exposure of breast tissue to ovarian hormones that drive the kinetics of breast tissue stem cells, resulting in carcinogenesis [38]. Dividing cells are particularly susceptible to alterations in DNA synthesis, DNA repair, and DNA methylation.

These biologic and physiologic effects of sex hormones are controlled by hormone receptors, the expression of which is regulated by the methylation status of their promoter regions [33-35].

On the other hand, steroid hormones may influence the epigenetic blue print of methylation of certain genes and consequently activate or inactivate gene expression [39]. Even though MBD2 might be involved in the epigenetic regulation of steroid hormone receptor gene expression, MBD2 itself could also be affected by steroid hormones. It is possible that MBD2 plays different roles in breast carcinogenesis when hormone levels dramatically change. Our findings support this speculation in that we found a significantly protective role of MBD2 variants in premenopausal women but no significant associations in postmenopausal women.

There are limitations to the present study. Only Caucasian women were included in the study, and so hypotheses must be further examined in multi-ethnic groups. In addition, the sample sizes of our study limit the analyses to explore other potential risk factors. Traditional risk factors such as parity and family history of cancer did not differ between cases and controls, which could also be due to the sample sizes. Inaccurate recall may affect our assessment of family history of cancer as well.

## Conclusion

These findings imply a potential link between DNA methylation processes and hormonal expression. Although large molecular epidemiologic studies are warranted to further examine associations between *MBD2 *polymorphisms and breast cancer in multi-ethnic groups, this study suggests that genetic variations in methylation related genes may serve as a promising biomarker in risk estimate of breast cancer.

## Abbreviations

bp = base pairs; CI = confidence interval; MBD = methyl-CpG-binding domain; OR = odds ratio; PCR = polymerase chain reaction; SNP = single nucleotide polymorphism.

## Competing interests

The authors declare that they have no competing interests.

## Authors' contributions

YZ designed the study and drafted the manuscript. HNB carried out the genotyping analysis. YZ and TRH performed the statistical analysis. TZ is the principal investigator for the parent case–control study and participated in the design of the present project and manuscript preparation. All authors read and approved the final manuscript.

## Figures and Tables

**Table 1:** Distributions of selected characteristics by case–control status in Caucasians

Variable	Cases (*n *= 393)	Controls (*n *= 436)	*P*
Age at menarche (years)			
<11	23 (5.9)	29 (6.7)	
11–12	152 (39.2)	168 (38.6)	
≥ 12	213 (54.9)	238 (54.7)	0.907
Menopausal status			
Premenopausal	88 (22.4)	146 (33.5)	
Postmenopausal	305 (77.6)	290 (66.5)	0.000
Age at menopause (years)			
<44	76 (25.8)	91 (32.3)	
44–49	105 (35.6)	87 (30.9)	
≥ 49	114 (38.6)	104 (36.8)	0.202
Lifetime lactation (months)			
0	242 (61.6)	260 (59.6)	
1–5	50 (12.7)	61 (14.0)	
6–15	56 (14.3)	59 (13.5)	
≥ 15	45 (11.4)	56 (12.9)	0.853
Family history of breast cancer in first-degree relatives			
Yes	94 (24.0)	98 (22.5)	
No	299 (76.0)	338 (77.5)	0.623
Body mass index (kg/m^2^)			
<25.0	72 (18.3)	77 (17.7)	
25.0–29.9	100 (25.5)	110 (25.2)	
≥ 30.0	221 (56.2)	249 (57.1	0.960
Fat intake			
Low	120 (31.0)	141 (33.2)	
Medium	132 (34.1)	143 (33.6)	
High	135 (34.9)	141 (33.2)	0.786
Cigarette smoking			
Never	169 (43.0)	196 (45.0)	
Ever	224 (57.0)	239 (55.0)	0.534
Alcohol consumption			
Never	66 (19.2)	64 (16.9)	
Ever	277 (80.8)	315 (83.1)	0.411
Annual income			
Low	126 (39.0)	125 (33.4)	
Medium	121 (37.5)	133 (35.6)	
High	76 (23.5)	116 (31.0)	0.074
Study site			
New Haven County	266 (67.7)	282 (64.7)	
Tolland County	127 (32.3)	154 (35.3)	0.361
Live births			
0	46 (11.95)	66 (15.46)	
1–2	200 (51.95)	202 (47.31	
≥ 2	139 (36.10)	159 (37.24)	0.252

Note that missing data for each characteristic were excluded from the analyses. Values are expressed as *n *(%).

**Table 2:** *MBD2 *genotypes, menopausal status and breast cancer risk in Caucasians

*MBD2 *genotype	Cases (*n *= 393)	Controls (*n *= 436)	Crude OR (95% CI)	OR^a ^(95% CI)
**SNP 1 (rs1259938)**				
All women				
G/G	210 (53.4)	226 (51.8)	1.00	1.00
G/A	158 (40.2)	179 (41.1)	0.95 (0.71–1.27)	0.94 (0.70–1.26)
A/A	25 (6.4)	31 (7.1)	0.86 (0.49–1.52)	0.86 (0.49–1.54)
G/A + A/A	183 (46.6)	210 (48.2)	0.94 (0.71–1.23)	0.94 (0.71–1.24)
Premenopausal women				
G/G	59 (67.1)	69 (47.3)	1.00	1.00
G/A	25 (28.4)	65 (44.5)	0.43 (0.24–0.77)	0.40 (0.22–0.74)
A/A	4 (4.6)	12 (8.2)	0.39 (0.12–1.26)	0.39 (0.12–1.29)
G/A + A/A	29 (33.0)	77 (52.7)	0.42 (0.24–0.73)	0.41 (0.23–0.72)
Postmenopausal women				
G/G	151 (49.5)	157 (54.1)	1.00	1.00
G/A	133 (43.6)	114 (39.3)	1.24 (0.88–1.74)	1.24 (0.88–1.75)
A/A	21 (6.9)	19 (6.6)	1.16 (0.59–2.27)	1.15 (0.58–2.29)
G/A + A/A	154 (50.5)	134 (45.9)	1.23 (0.90–1.70)	1.24 (0.90–1.72)
**SNP 2 (rs609791)**				
All women				
C/C	243 (63.9)	260 (61.6)	1.00	1.00
C/G	120 (31.6)	140 (33.2)	0.92 (0.68–1.24)	0.92 (0.68–1.24)
G/G	17 (4.5)	22 (5.2)	0.83 (0.43–1.59)	0.85 (0.44–1.64)
C/G + G/G	237 (36.1)	162 (38.4)	0.90 (0.68–1.21)	0.91 (0.68–1.22)
Premenopausal women				
C/C	60 (70.6)	80 (56.3)	1.00	1.00
C/G	20 (23.5)	55 (38.7)	0.48 (0.26–0.89)	0.47 (0.26–0.88)
G/G	5 (5.9)	7 (5.0)	0.95 (0.29–3.15)	1.02 (0.30–3.49)
C/G + G/G	25 (29.4)	62 (43.7)	0.54 (0.30–0.95)	0.54 (0.30–0.96)
Postmenopausal women				
C/C	183 (62.0)	180 (64.3)	1.00	1.00
C/G	100 (33.9)	85 (30.4)	1.16 (0.81–1.65)	1.15 (0.80–1.64)
G/G	12 (4.1)	15 (5.3)	0.79 (0.36–1.73)	0.80 (0.36–1.76)
C/G + G/G	112 (38.0)	100 (35.7)	1.10 (0.78–1.55)	1.10 (0.78–1.55)

Values are expressed as *n *(%). ^a^Adjusted for age (as a continuous variable), body mass index (<25 kg/m^2^, 25–29.99 kg/m^2^, >29.99 kg/m^2^), family history of breast cancer in first-degree relatives, menopausal status, family income (tertiles based on distribution of controls), lifetime months of breast feeding (never, 1–5, 6–15, ≥ 15 months) and study site. CI, confidence interval; OR, odds ratio.

**Table 3:** *MBD2 *Haplotypes, menopausal status and breast cancer risk in Caucasians

*MBD2 *Haplotypes	Frequency in cases	Frequency in controls	Crude OR (95% CI)	OR^a ^(95% CI)
All women				
GC	69.22%	66.90%	Reference	Reference
GG	4.81%	5.56%	0.84 (0.53–1.31)	0.82 (0.52–1.29)
AC	11.17%	11.70%	0.92 (0.68–1.26)	0.92 (0.67–1.25)
AG	14.81%	15.84%	0.90 (0.69–1.19)	0.90 (0.68–1.19)
Premenopausal women				
GC	77.65%	63.29%	Reference	Reference
GG	5.29%	5.59%	0.77 (0.33–1.80)	0.77 (0.33–1.81)
AC	6.47%	12.59%	0.42 (0.21–0.85)	0.40 (0.20–0.83)
AG	10.59%	18.53%	0.47 (0.26–0.83)	0.47 (0.26–0.84)
Postmenopausal women				
GC	66.83%	68.75%	Reference	Reference
GG	4.67%	5.54%	0.87 (0.51–1.47)	0.84 (0.49–1.44)
AC	12.50%	11.25%	1.14 (0.80–1.64)	1.14 (0.79–1.65)
AG	16.00%	14.46%	1.14 (0.82–1.58)	1.13 (0.81–1.57)

Values are expressed as *n *(%). ^a^Adjusted for age (as a continuous variable), body mass index (<25 kg/m^2^, 25–29.99 kg/m^2^, >29.99 kg/m^2^), family history of breast cancer in first-degree relatives, menopausal status, family income (tertiles based on distribution of controls), lifetime months of breast feeding (never, 1–5, 6–15, ≥15 months) and study site. CI, confidence interval; OR, odds ratio.

## References

[B1] Jones PA (2003). Epigenetics in carcinogenesis and cancer prevention. Ann NY Acad Sci.

[B2] Razin A (1998). CpG methylation, chromatin structure and gene silencing-a three-way connection. EMBO J.

[B3] Davey C, Pennings S, Allan J (1997). CpG methylation remodels chromatin structure *in vitro*. J Mol Biol.

[B4] Antequera F, Boyes J, Bird A (1990). High levels of *de novo* methylation and altered chromatin structure at CpG islands in cell lines. Cell.

[B5] Cross SH, Meehan RR, Nan X, Bird A (1997). A component of the transcriptional repressor MeCP1 shares a motif with DNA methyltransferase and HRX proteins. Nat Genet.

[B6] Hendrich B, Bird A (1998). Identification and characterization of a family of mammalian methyl-CpG binding proteins. Mol Cell Biol.

[B7] Nan X, Ng HH, Johnson CA, Laherty CD, Turner BM, Eisenman RN, Bird A (1998). Transcriptional repression by the methyl-CpG-binding protein MeCP2 involves a histone deacetylase complex. Nature.

[B8] Patra SK, Patra A, Zhao H, Carroll P, Dahiya R (2003). Methyl-CpG-DNA binding proteins in human prostate cancer: expression of CXXC sequence containing MBD1 and repression of MBD2 and MeCP2. Biochem Biophys Res Commun.

[B9] Schlegel J, Guneysu S, Mennel HD (2002). Expression of the genes of methyl-binding domain proteins in human gliomas. Oncol Rep.

[B10] Hendrich B, Abbott C, McQueen H, Chambers D, Cross S, Bird A (1999). Genomic structure and chromosomal mapping of the murine and human Mbd1, Mbd2, Mbd3, and Mbd4 genes. Mamm Genome.

[B11] Snape A (2000). MBDs mediate methylation, deacetylation and transcriptional repression. Trends Genet.

[B12] Wade PA (2001). Methyl CpG-binding proteins and transcriptional repression. Bioessays.

[B13] Bhattacharya SK, Ramchandani S, Cervoni N, Szyf M (1999). A mammalian protein with specific demethylase activity for mCpG DNA. Nature.

[B14] Ng HH, Zhang Y, Hendrich B, Johnson CA, Turner BM, Erdjument-Bromage H, Tempst P, Reinberg D, Bird A (1999). MBD2 is a transcriptional repressor belonging to the MeCP1 histone deacetylase complex. Nat Genet.

[B15] Wade PA, Gegonne A, Jones PL, Ballestar E, Aubry F, Wolffe AP (1999). Mi-2 complex couples DNA methylation to chromatin remodelling and histone deacetylation. Nat Genet.

[B16] Billard LM, Magdinier F, Lenoir GM, Frappart L, Dante R (2002). MeCP2 and MBD2 expression during normal and pathological growth of the human mammary gland. Oncogene.

[B17] Lin X, Nelson WG (2003). Methyl-CpG-binding domain protein-2 mediates transcriptional repression associated with hypermethylated GSTP1 CpG islands in MCF-7 breast cancer cells. Cancer Res.

[B18] Kanai Y, Ushijima S, Nakanishi Y, Hirohashi S (1999). Reduced mRNA expression of the DNA demethylase, MBD2, in human colorectal and stomach cancers. Biochem Biophys Res Commun.

[B19] Zhu Y, Spitz MR, Zhang H, Grossman HB, Frazier ML, Wu X (2004). Methyl-CpG-binding domain 2: a protective role in bladder carcinoma. Cancer.

[B20] Patra SK, Patra A, Zhao H, Dahiya R (2002). DNA methyltransferase and demethylase in human prostate cancer. Mol Carcinog.

[B21] Zheng T, Holford TR, Mayne ST, Tessari J, Ward B, Carter D, Owens PH, Boyle P, Dubrow R, Archibeque-Engle S (2000). Risk of female breast cancer associated with serum polychlorinated biphenyls and 1,1-dichloro-2,2'-bis(p-chlorophenyl)ethylene. Cancer Epidemiol Biomarkers Prev.

[B22] Singletary SE, Allred C, Ashley P, Bassett LW, Berry D, Bland KI, Borgen PI, Clark GM, Edge SB, Hayes DF (2003). Staging system for breast cancer: revisions for the 6th edition of the AJCC Cancer Staging Manual. Surg Clin North Am.

[B23] Single Nucleotide Polymorphism Database. http://www.ncbi.nlm.nih.gov/SNP/index.html.

[B24] Pesole G, Mignone F, Gissi C, Grillo G, Licciulli F, Liuni S (2001). Structural and functional features of eukaryotic mRNA untranslated regions. Gene.

[B25] Stephens M, Smith NJ, Donnelly P (2001). A new statistical method for haplotype reconstruction from population data. Am J Hum Genet.

[B26] Woodcock DM, Linsenmeyer ME, Doherty JP, Warren WD (1999). DNA methylation in the promoter region of the p16 (CDKN2/MTS-1/INK4A) gene in human breast tumours. Br J Cancer.

[B27] Herman JG, Merlo A, Mao L, Lapidus RG, Issa JP, Davidson NE, Sidransky D, Baylin SB (1995). Inactivation of the CDKN2/p16/MTS1 gene is frequently associated with aberrant DNA methylation in all common human cancers. Cancer Res.

[B28] Esteller M, Corn PG, Urena JM, Gabrielson E, Baylin SB, Herman JG (1998). Inactivation of glutathione S-transferase P1 gene by promoter hypermethylation in human neoplasia. Cancer Res.

[B29] Magdinier F, Billard LM, Wittmann G, Frappart L, Benchaib M, Lenoir GM, Guerin JF, Dante R (2000). Regional methylation of the 5' end CpG island of BRCA1 is associated with reduced gene expression in human somatic cells. FASEB J.

[B30] Dobrovic A, Simpfendorfer D (1997). Methylation of the *BRCA1* gene in sporadic breast cancer. Cancer Res.

[B31] Friedman LS, Ostermeyer EA, Lynch ED, Szabo CI, Anderson LA, Dowd P, Lee MK, Rowell SE, Boyd J, King MC (1994). The search for *BRCA1*. Cancer Res.

[B32] Fishman J, Osborne MP, Telang NT (1995). The role of estrogen in mammary carcinogenesis. Ann N Y Acad Sci.

[B33] Ottaviano YL, Issa JP, Parl FF, Smith HS, Baylin SB, Davidson NE (1994). Methylation of the estrogen receptor gene CpG island marks loss of estrogen receptor expression in human breast cancer cells. Cancer Res.

[B34] Archey WB, McEachern KA, Robson M, Offit K, Vaziri SA, Casey G, Borg A, Arrick BA (2002). Increased CpG methylation of the estrogen receptor gene in *BRCA1*-linked estrogen receptor-negative breast cancers. Oncogene.

[B35] Widschwendter M, Berger J, Hermann M, Muller HM, Amberger A, Zeschnigk M, Widschwendter A, Abendstein B, Zeimet AG, Daxenbichler G (2000). Methylation and silencing of the retinoic acid receptor-beta2 gene in breast cancer. J Natl Cancer Inst.

[B36] Thompson PA, Shields PG, Freudenheim JL, Stone A, Vena JE, Marshall JR, Graham S, Laughlin R, Nemoto T, Kadlubar FF (1998). Genetic polymorphisms in catechol-O-methyltransferase, menopausal status, and breast cancer risk. Cancer Res.

[B37] Lee HP, Gourley L, Duffy SW, Esteve J, Lee J, Day NE (1992). Risk factors for breast cancer by age and menopausal status: a case-control study in Singapore. Cancer Causes Control.

[B38] Henderson BE, Ross R, Bernstein L (1988). Estrogens as a cause of human cancer: the Richard and Hinda Rosenthal Foundation award lecture. Cancer Res.

[B39] Jost JP, Saluz HP (1993). Steroid hormone dependent changes in DNA methylation and its significance for the activation or silencing of specific genes. EXS.

